# Mutations that prevent or mimic persistent post-translational modifications of the histone H3 globular domain cause lethality and growth defects in *Drosophila*

**DOI:** 10.1186/s13072-016-0059-3

**Published:** 2016-02-29

**Authors:** Hillary K. Graves, Pingping Wang, Matthew Lagarde, Zhihong Chen, Jessica K. Tyler

**Affiliations:** Department of Epigenetics and Molecular Carcinogenesis, University of Texas MD Anderson Cancer Center, Houston, TX 77030 USA; Department of Pathology and Laboratory Medicine, Weill Cornell Medical College, New York, NY 10065 USA

**Keywords:** Histone post-translational modifications, *Drosophila*, Imaginal discs, Histone H3 globular domain, Development

## Abstract

**Background:**

Understanding the function of histone post-translational modifications is the key to deciphering how genomic activities are regulated. Among the least well-understood histone modifications in vivo are those that occur on the surface of the globular domain of histones, despite their causing the most profound structural alterations of the nucleosome in vitro. We utilized a *Drosophila* system to replace the canonical histone genes with mutated histone transgenes.

**Results:**

Mutations predicted to mimic or prevent acetylation on histone H3 lysine (K) 56, K115, K122, and both K115/K122, or to prevent or mimic phosphorylation on H3 threonine (T) 118 and T80, all caused lethality, with the exception of *K122R* mutants. *T118* mutations caused profound growth defects within wing discs, while *K115R*, *K115Q*, *K56Q*, and the *K115/K122* mutations caused more subtle growth defects. The H3 *K56R* and *H3 K122R* mutations caused no defects in growth, differentiation, or transcription within imaginal discs, indicating that H3 K56 acetylation and K122 acetylation are dispensable for these functions. In agreement, we found the antibody to H3 K122Ac, which was previously used to imply a role for H3 K122Ac in transcription in metazoans, to be non-specific in vivo.

**Conclusions:**

Our data suggest that chromatin structural perturbations caused by acetylation of K56, K115, or K122 and phosphorylation of T80 or T118 are important for key developmental processes.

**Electronic supplementary material:**

The online version of this article (doi:10.1186/s13072-016-0059-3) contains supplementary material, which is available to authorized users.

## Background

All of the activities of the genome are regulated by its packaging into chromatin, by inherently limiting access of cellular machineries to the DNA. The repeating unit of chromatin is the nucleosome, comprising approximately 146 bp of DNA wound approximately twice around two copies of each of the H2A, H2B, H3, and H4 histone proteins. The histone proteins are subject to many different types of post-translational modifications (PTMs) including phosphorylation (p), methylation (me), acetylation (Ac), and ubiquitination (ub) [1]. Understanding the function of histone PTMs is the key to deciphering how genomic activities are regulated. PTMs on the N-terminal tail of histones are not predicted to change the nucleosome structure per se but generally create binding sites for effector proteins [2]. By contrast, PTMs on the histone globular domains (the area inside of the nucleosomal DNA) can directly loosen the nucleosome structure if the residue normally mediates histone–DNA interactions within a nucleosome. The ability of histone globular domain PTMs to directly change the nucleosome structure is well established biochemically [3], but their functional roles in vivo are poorly appreciated. 

Of the histone H3 globular domain modifications, acetylation of lysine 56 (K56Ac) which is conserved from yeast to humans is the best studied. H3 K56 resides in the N-terminal alpha helix at the point where DNA enters and exits the nucleosome and K56 normally binds to DNA. Not surprisingly therefore, acetylation of H3 K56 leads to an increased likelihood of the DNA unwrapping from the entry and exit point of the nucleosome [4, 5]. It has been shown functionally that H3 K56Ac promotes the assembly and disassembly of nucleosomes in yeast, leading to efficient DNA repair, replication, and transcription [6–8]. Direct study of H3 K56Ac function in metazoan cells has been limited to immunolocalization, where H3 K56Ac levels in flies and humans increase at regions undergoing transcription and DNA repair, suggesting that H3 K56Ac may have similar roles in metazoans as it does in yeast [9–11]. However, the levels of H3 K56Ac in mammalian cells is much lower than in yeast and to date the function of H3 K56Ac has not been directly examined in vivo beyond yeast.

The family of histone globular domain PTMs also includes three modifications of amino acids that mediate histone–DNA interactions at the nucleosome dyad [12]. The dyad is the point at which DNA is only wrapped once around the nucleosome and is therefore most sensitive to perturbation of the histone–DNA contacts. These dyad PTMs are phosphorylation of T118 (T118p) and acetylation of K115 (K115Ac) and K122 (K122Ac) and were first identified by mass spectrometry analysis of bovine histones, where they often occurred together on the same peptide [13]. These three dyad PTMs have subsequently been identified in human cells [11, 14], in flies for T118p (Wike and Tyler, unpublished observation), and in fission yeast for K122Ac [15]. On reconstituted nucleosomes, these three modifications each promote chromatin disassembly, while H3 T118p additionally promotes nucleosome mobility [16, 17]. H3 K122Ac promotes transcription on reconstituted chromatin templates [15] and movement of the mismatch repair proteins MSH2-MSH6 along the template by facilitating histone removal from the DNA [18]. Chromatin immunoprecipitation (ChIP) analysis of H3 K122Ac in mammalian cells found it to reside at active enhancers, which is consistent with a role for this modification in transcriptional activation [15]. Mutation of the H3 K122 and H3 K115 residues in yeast causes DNA damage sensitivity and defects in transcriptional induction and silencing [19, 20], and it is possible therefore that acetylation may be used by metazoan cells to regulate transcription and repair in a similar manner by altering the chromatin structure. The existence of H3 K115Ac has not yet been described in yeast and no specific antibodies exist to it, limiting its further analysis. As such, the function or importance of H3 K115Ac in cells is unknown. H3 T118p which causes the most disruption to the nucleosome in vitro [16] occurs specifically during mitosis in flies, worms, and human cells, and it promotes progression through mitosis in metazoans [21].

Other PTMs within the globular domains of histones may influence interactions between nucleosomes. H3 T80p occurs on the lateral surface of the nucleosome and was originally characterized using an antibody [22] that transpired to preferentially recognize H3 S10p [23]. Upon re-examination with a specific H3 T80p antibody, it was shown that H3 T80p occurs only in specific phases of mitosis and overexpression of mutants that disrupt H3 T80 in mammalian cells leads to mitotic defects [23]. Its location of the lateral side of the nucleosome and its specific interaction with other histones led us to propose that T80p promotes chromatin compaction during mitosis by facilitating nucleosome–nucleosome interactions [23]. The importance of H3 T80p in development is unknown.

Although genetic analysis of histone mutants is commonly performed in yeast where there are only two copies of each histone gene, in vertebrates, canonical histone genes are encoded by multiple gene units of between 10 and 400 copies that are distributed across the genome making gene knockouts or knockdown by RNAi implausible [24]. Therefore, functional studies of histone modifications beyond yeast have typically relied on the overexpression of histones bearing mutations that mimic/prevent PTMs in the context of endogenous histone gene expression. This only yields information if the mutation has a dominant negative function, which is rarely the case. These technical hurdles have led to many correlative, rather than functional, studies of histone modifications in metazoans [25]. To overcome these technical problems, genetic approaches were developed in *Drosophila* wherein the locus containing approximately 200 genes expressing the canonical histones is replaced by 12 copies of each histone gene supplied on transgenes [26, 27]. When these histone transgenes bearing a specific mutation are re-introduced into the flies, all of the histone protein within the animal will carry the mutation. This method for histone replacement in flies has recently been used to examine the importance of H3 K4me, H3 K27me, H3 K36me, H2Aub, and H4 K20me modifications [26–29]. To examine the function of histone modifications that are essential for viability, this system has been adapted for clonal analysis of the effect of histone mutations on cellular processes in imaginal tissues [28–31]. Unexpected results were found in flies unable to methylate H3 K4 [28]. H3 K4 methylation has long been assumed to help regulate gene expression from studies in yeast and its occurrence on active genes [1]. However, flies where all histone H3 carried the K4A mutation had no obvious defects in transcription [28], indicating that its perceived role in transcription from localization correlation studies was likely to have been overinterpreted. This work highlighted the importance of functional analysis of histone PTMs in metazoans via the use of mutations in the absence of wild-type endogenous histones.

*Drosophila* provides a unique system to examine the biological significance of histone H3 globular domain PTMs in a multicellular organism. Here we made mutations predicted to prevent or mimic the acetylation of histone H3 residues K56, K115, and K122 as well as the phosphorylation of histone H3 T80 and T118 to examine the role of their modification on development in *Drosophila*. We show that mutation of any one of these residues, with the exception of the *K122R* mutation, causes lethality, and that all the mutations, with the exception of those affecting H3 T80 and H3 K122, cause growth defects within the wing disc. However, none of these residues are essential for either transcription or differentiation within the contexts we assayed. This study provides the first in vivo analysis of the role of post-translational modification of the histone H3 globular domain in development.

## Results

### Mutation of H3 residues K56, T80, K115, T118, and K122 results in lethality in *Drosophila*

**Fig. 1:**
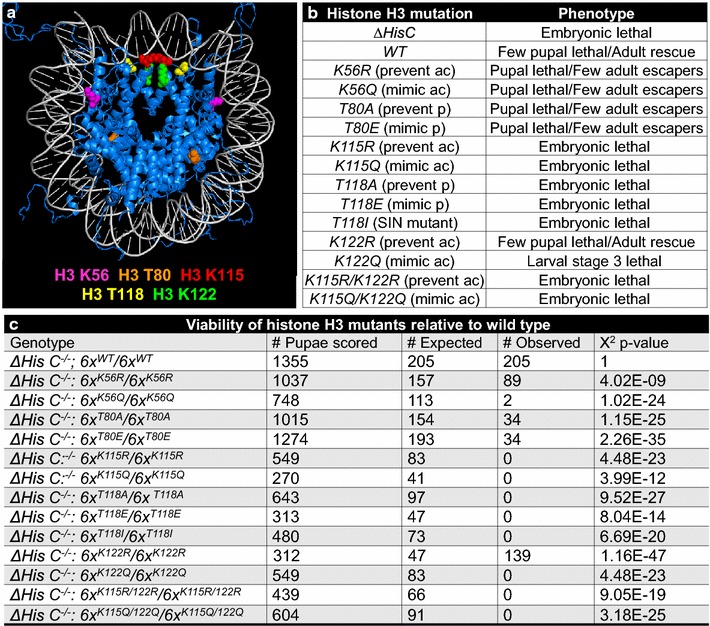
Mutations in residues within the globular domain of histone H3 cause lethality. **a** *Ribbon structure* of a nucleosome with histones represented in *blue* and globular domain histone H3 modifications shown in the *colors* indicated. H3 K115Ac, H3 T118p, and H3 K122Ac are located at the dyad axis, H3 K56Ac is located at the DNA entry/exit point, and H3 T80p occurs at the lateral surface of the nucleosome. **b** Table indicating the developmental lethal phase of the indicated *Drosophila* strains expressing no canonical histones (*ΔHisC*) or wild type (WT) or the indicated H3 mutants expressed from *12xHisGU* ^*VK33*,*27*^ transgenes. **c** Chi-squared analysis of the expected and observed frequency of emergence of flies from pupae from the progeny from the self-cross *yw*; *ΔHisC FRT40A*; *6xHisGU* ^*VK33*,*27*^ *WT*/*SM5*^*TM6B* or *yw*; *ΔHisC FRT40A*; *H3mutant*/*SM5*^*TM6B*. Some *ΔHisC* ^−*/*−^; *12xHisGU* ^*VK33*,*27*^ *WT* are pupal lethal, so the *p* values indicated for the *H3* mutants are normalized to *wild type*

Given that the modifications H3 K56Ac, T80p, K115Ac, T118p, and K122Ac occur at histone surfaces that normally mediate histone–DNA interactions or potentially nucleosome–nucleosome interactions (Fig. 1a), we were interested in determining their importance in a metazoan using the *Drosophila* system. The *Drosophila* canonical histone genes are located in a single cluster (*HisC*) on chromosome II that contains ~100 repeats of the histone gene unit (*HisGU*), each having one gene copy of the four canonical core histones H2A, H2B, H3, and H4, and the linker histone H1 [27, 32]. Animals homozygous for a chromosomal deletion that removes the histone gene cluster (*ΔHisC*) die at the blastoderm stage, after exhaustion of the supply of maternally loaded histones [26]. Transgene cassettes providing 12 copies of the wild-type *HisGU* rescue *ΔHisC* homozygotes into viable adults. Accordingly, we mutated each of the 12 copies of the *H3* gene on transgenes and introduced them into *Drosophila* lacking endogenous *H3*, such that only mutant *H3* was expressed [26] (for details, see “Methods” section).

For the acetylatable lysines, we mutated them to glutamine (Q) to mimic acetylation and arginine (R) to prevent acetylation. Given that K115Ac and K122Ac often co-occur on the same histone peptide [11, 13], we also mutated them together. For the phosphorylated threonines, we mutated them to glutamic acid (E) to mimic phosphorylation and to alanine (A) to prevent phosphorylation. We also mutated T118 to an isoleucine (T118I), which was previously identified in *S. cerevisiae* as a dominant Snf2-independent (SIN) allele [33]. The SIN H3 T118I substitution allows nucleosomes to slide along the DNA without the need for SWI/SNF [34]. H3 T118I is structurally predicted to be a better mimic of the ability of T118 phosphorylation to repel DNA, due to its being more rigid than E, so more like phosphorylation, while still being bulky. Compared to control animals bearing wild-type (*WT*) *H3*, animals expressing the mutant *H3* transgenes did not survive to adulthood and died at various stages of development (Fig. 1b). Mutations affecting T118 and K115 were lethal at the embryonic stage, *K122Q* mutants were lethal at larval stage L3, *K122R* mutants rescued better than WT, and those affecting T80 and K56 did not display lethality until the pharate adult stage. Chi-squared analyses show that the difference in frequency of emergence of flies from pupae for the *WT* and mutant flies is statistically significant (Fig. 1c). These results indicate that histone H3 residues K56, T80, K115, T118, and K122, and potentially their post-translational modification, are essential for *Drosophila* development, with each functioning at different stages of development.

### Mutation of H3 K56, K115, and T118, but not H3 T80 or K122 disrupts normal growth within imaginal discs

Given that mutations affecting the globular domain of histone H3 were lethal, we created distinct regions of WT and mutant histone expression within the imaginal discs of flies using the FLP/FRT system. In this method, homozygous mutant *ΔHisC* cells, or “mutant clones,” can be induced in particular tissues using a tissue-specific or temperature-sensitive flippase in an animal that is otherwise heterozygous for the *ΔHisC* mutation [28, 30, 35]. The *12xHisGU* transgenes are expressed in all cells, but the amount of endogenous histones expressed differs between cells. Specifically, lighter GFP marks the cells that are heterozygous for the *ΔHisC* mutation. Mutant clones lacking endogenous histones are marked by the absence of GFP and the adjacent clones with more intense GFP mark twin spots that are homozygous for wild-type *HisC*. Because the twin spot results from the same homologous recombination event that generated the adjacent mutant clones, they are useful for indicating the size of the clone that would be expected if the histone mutation had no effect on cell growth or viability.

**Fig. 2:**
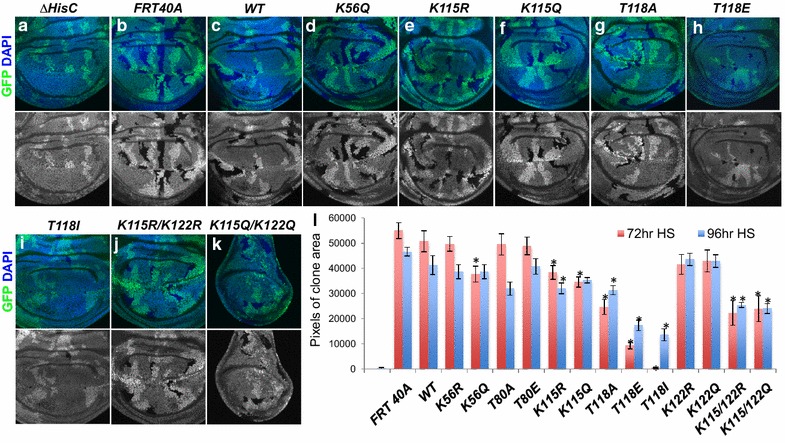
Mutation of H3 K115, T118 and K115/K122 and K56Q causes growth defects in imaginal discs. **a**–**k** ×40 images of wing imaginal discs with GFP-negative mutant clones generated using *heat-shock FLP* at 72 h AEL. *Merged images* show the nuclear marker DAPI and GFP^+^ and GFP^−^ regions demarcate histone wild-type cells and histone mutant cells, respectively. *Grayscale* images are the GFP channels individually, shown for contrast. Note the smaller size of most GFP^−^ mutant areas compared to their GFP^++^ twin spot. *ΔHisC* mutant clones cannot survive. **l** *Graph* representing the quantification of average clone area per ×40 wing disc image. *Asterisk* represents significance at *p* < 0.05, which was calculated using a two-sample *t* test with *wild type* as the reference group

To determine whether modification of H3 residues K56, T80, K115, T118, and K122 is required for cell cycle progression in *Drosophila*, we induced clones at both 72 and 96 h after egg laying (AEL), using *heat-shockFLP*, and then measured the area occupied by clones in wing imaginal discs dissected from wandering L3 larvae at ~144 h AEL (see “Methods” section). Indeed, several of the histone modification mutants led to mutant clones that were visibly smaller than their twin spots, indicating a growth defect. Importantly, *ΔHisC*^−/−^ clones rescued by *12x WT* transgenes grew approximately as well as the positive control *FRT40A* chromosome that is wild type for the endogenous *HisC* locus, whereas the *ΔHisC*^−/−^ mutant clones barely grew (Fig. 2a–c, l). Mutant clone areas per imaginal disc were quantitated, revealing that mutant histone *H3 K56Q*, *K115R*, *K115Q*, *T118A*, *T118E*, *T118I*, *K115R*/*K122R*, and *K115Q*/*K122Q* clones all occupied significantly less area than controls (Fig. 2d–k, l), suggesting a longer or dysregulated cell cycle. In fact, the *T118I* mutant clones occupied almost as little area as the *ΔHisC*^−/−^ mutant clones (Fig. 2a, i, l). By contrast, the clones expressing only H3 K56R, T80A, T80E, K122R, and K122Q occupied an approximately equivalent area to those expressing wild-type histones, indicating that dynamic T80p, K56Ac, and K122Ac do not play a key role in cell growth during development.

**Fig. 3:**
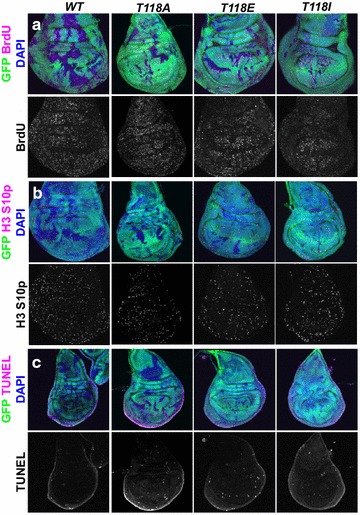
There is no overt changes in cell cycle or apoptosis markers in *H3 T118* mutant clones. Wing imaginal discs with GFP-negative mutant clones generated using *Ubx*-*FLP*. **a** *Merged images* show the nuclear marker DAPI in *blue*, BrdU in *magenta*, and GFP^+^ and GFP^−^ regions demarcate histone wild-type cells and histone mutant cells, respectively. *Grayscale* images are the individual BrdU channels. Within each mutant, we looked at GFP^−^ clones within the zone of non-proliferation of the wing disc (see Additional file 1: Fig. S1) in search of consistent increases in BrdU incorporation. Conversely, we looked at GFP^−^ clones outside of the zone of non-proliferation to determine whether there was a consistent decrease of BrdU incorporation compared to their neighboring GFP^+^ control cells. **b** *Merged images* show the DNA marker DAPI in *blue*, H3 S10p in *magenta*, and GFP^+^ and GFP^−^ regions demarcate histone wild-type cells and histone mutant cells, respectively. *Grayscale* images are the individual H3 S10p channels. Within each mutant, we looked at GFP^−^ clones within the zone of non-proliferation of the wing disc to determine whether there was a consistent increase of H3 S10p incorporation, which might indicate an increase of mitotic cells. Conversely, we looked at GFP^−^ clones outside of the zone of non-proliferation to determine whether there was a consistent decrease of H3 S10p incorporation compared to their neighboring GFP^+^ control cells. **c** *Merged images* show the DNA marker DAPI in *blue*, TUNEL in *magenta*, and GFP^+^ and GFP^−^ regions demarcate histone wild-type cells and histone mutant cells, respectively. *Grayscale* images are the individual TUNEL channels. Within each mutant genotype, we looked at GFP^−^ clones outside the edges of the disc to determine whether there was a consistent increase of TUNEL compared to their neighboring GFP^+^ control cells

To identify the cause of the poor growth phenotype caused by the histone H3 mutations, we assayed for differences in cell cycle and apoptotic markers in mosaic wing discs with clones of cells expressing mutant histones. These clones were generated using *ubx*-*FLP*, which is expressed continuously starting from embryogenesis such that many overlapping clones and twin spots are generated. By specifically examining BrdU incorporation within the mutant clones, we did not notice any profound increase or decrease in the proportion of cells undergoing replication for any of the histone mutations (Fig. 3a; Additional file 1: Figure S1). To determine whether any of the histone mutations led to accumulation of cells in mitosis, which would be indicative of activation of the spindle assembly checkpoint or the DNA damage checkpoint, we examined H3 S10 phosphorylation. We did not notice an accumulation of cells with H3 S10p staining within any of the mutant histone clones, as compared to the frequency of cells with H3 S10p staining outside of the mutant histone clones (Fig. 3b; Additional file 2: Figure S2). As such, the mutation of the PTM sites within the globular domain of H3 does not lead to profound defects in either replication or mitosis.

Lack of growth can be due to reduced cell growth or more cell death. Therefore, we used TUNEL analysis to assay for cell death in mosaic wing discs. *GMR*-*hid*, which is a transgenic fly that expresses the apoptosis-regulating gene *hid* in the eye, serves as a positive control for increased apoptosis in the posterior region of the eye disc [36] (Additional file 3: Figure S3). By contrast, there was no obvious decrease or increase in the number of cells undergoing apoptosis within any of the histone mutant clones compared to wild type (Fig. 3c; Additional file 3: Figure S3). Given that apoptosis was not increased and cell arrest was not apparent within the mutant clones, yet the clones were smaller, we suggest that the cell cycle defect/cell death caused by these histone mutations in imaginal discs is too subtle to be detected using the assays we have available.

During analysis of apoptosis, we noticed that the *H3 K115Q*/*K122Q* mosaic discs displayed a generalized increase in cell death within the disc proper (Additional file 3: Figure S3). The increase in general apoptosis in the *H3 K115Q*/*K122Q* mosaic discs suggests that the H3 K115Q/K122Q histones may have a dominant effect on tissue growth. The *H3 K115Q*/*K122Q* mutant also had a smaller adult mosaic eye, consistent with it having a negative influence on growth on the entire disc.

### Acetylation of H3 residues K56 and K122 is not required for transcription of several important developmental genes in imaginal discs, and the previously used antibody to K122Ac is non-specific in flies and mammalian cells

**Fig. 4:**
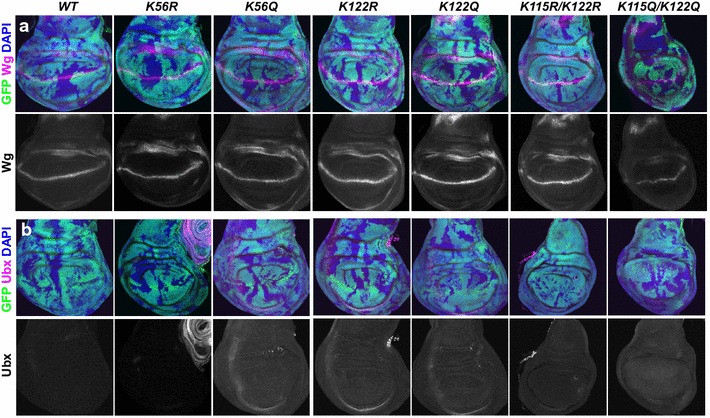
Markers of developmental signaling pathways are normal within cells that have mutations which prevent/mimic globular domain histone H3 modifications. Wing imaginal discs with GFP-negative mutant clones generated using *Ubx*-*FLP*. **a** *Merged images* show the DNA marker DAPI in *blue*, Wg in *magenta*, and GFP^+^ and GFP^−^ regions demarcate histone wild-type cells and histone mutant cells, respectively. *Grayscale* images are the individual Wg channels. **b** *Merged images* show the nuclear marker DAPI in *blue*, Ubx in *magenta*, and GFP^+^ and GFP^−^ regions demarcate histone wild-type cells and histone mutant cells, respectively. *Grayscale* images are the individual Ubx channels. Note that Ubx is expressed in the peripodial membrane of wing discs, tracheal cells that are accidently attached to the wing discs, as well as in leg discs, allowing for a positive control for the staining

The H3 K122Ac and H3 K56Ac PTMs within the globular domain of histone H3 have been suggested to regulate transcription by loosening the nucleosome structure, based on mutational studies in yeast and in vitro analyses, but this has not yet been tested in a metazoan in vivo. Theoretically, if proper transcriptional events have occurred, expression of developmental markers will be normal within mutant clones. If the wing has been properly patterned, cells at the dorsal/ventral boundary of the wing imaginal discs will express the protein wingless (Wg). We found that the Wg expression pattern was not detectably disrupted within imaginal wing discs in any of the mutant histone clones that expressed only the mutant histones (Fig. 4a; Additional file 4: Figure S4). As such, the absence of K56Ac, T80p, K115Ac, K122Ac, or T118p does not noticeably disrupt gene activation in developing wing imaginal discs.

Transcriptional repression is mediated through a tight chromatin structure. Given that the biochemical function of the H3 globular domain PTMs is to alter nucleosome structure or nucleosome packaging, we looked for defects in repression. We examined whether transcriptional repression was altered by the mutations that prevent or mimic the PTMs on K56, T80, K115, T118, and K122. We found that Ubx, a protein that is not normally expressed in wing imaginal discs, was properly repressed in all the histone mutant discs (Fig. 4b; Additional file 5: Figure S5). These results suggest that the modification of K56, T80, K115, T118, and K122 are not involved in transcriptional activation of Wg or repression of Ubx during developmental patterning of the wing.

**Fig. 5:**
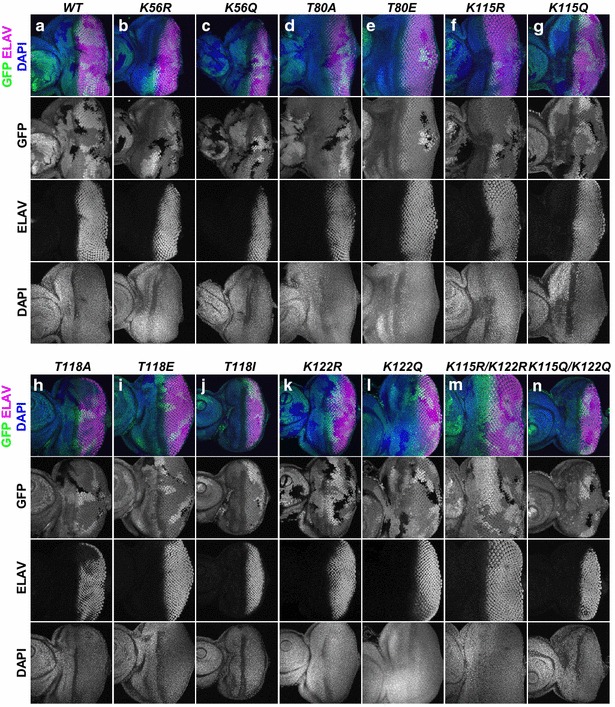
Differentiation, and therefore transcription, occurs normally within cells that have mutations which prevent/mimic globular domain histone H3 modifications. **a**–**n** Eye imaginal discs with GFP-negative mutant clones generated using *heat-shock* *FLP*. *Merged images* show the nuclear marker DAPI in *blue*, the neuronal marker ELAV in *magenta*, and GFP^+^ and GFP^−^ regions demarcate histone wild-type cells and histone mutant cells, respectively. *Grayscale* images are the indicated individual channels

In the eye imaginal disc, cells in the posterior region differentiate into photoreceptor neurons after a series of developmental signaling events [37]. To test whether differentiation occurs properly within the mutant cells, we examined the expression of the neuronal marker ELAV. We found that ELAV expression appeared normal at larval stage L3 in all of the mutants (Fig. 5); however, several of the mutations led to abnormal adult eye structures when clones were induced during development. These included rough eye and small eye phenotypes (Additional file 6: Figure S6), suggesting that cellular defects likely occur after pupation. However, it is important to keep in mind that because of the presence of white+ and yellow+ marker genes in the histone transgene cassette inserts, it is not possible to generate yellow− or white− marked *∆HisC* clones in adult tissues. Therefore, the genotype of the cells that form the differentiated structures (i.e., the eye) is not known and it is not known whether the rough eye phenotype is caused by abnormally differentiated mutant cells or by death of the mutant cells and inability of the surrounding healthy tissue to compensate. In summary, while H3 K56Ac and H3 K122Ac have been suggested to influence transcription, our data show that in vivo H3 K56Ac, H3 K115Ac, H3 K122Ac, H3 T80p, and H3 T118p do not appear to influence transcription or differentiation within the imaginal discs, while they are important for proper development.

**Fig. 6:**
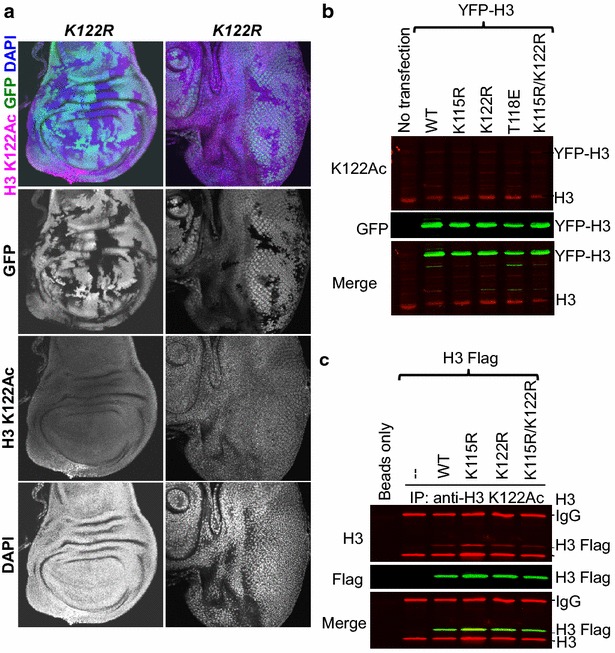
Commercial H3 K122Ac antibodies are not specific. **a** Wing and eye imaginal discs with GFP-negative mutant clones. *Merged images* show the nuclear marker DAPI in *blue*, H3 K122Ac in *magenta*, and GFP^+^ and GFP^−^ regions demarcate histone wild-type cells and histone mutant cells, respectively. *Grayscale* images are the individual H3 K122Ac channels. **b** Western blot analysis with the Abcam anti-H3 K122Ac antibody using acid-extracted histones collected from HEK293T cells transiently transfected with YFP-tagged WT or mutant histone H3 expression plasmid (H3.1 K115R, K122R, T118E, or K115R/K122R) shows that the anti-H3 K122Ac antibody non-specifically recognizes unmodified H3 and/or other modification(s) besides H3 K122Ac. **c** Immunoprecipitation (IP) analysis with the Abcam anti-H3 K122Ac antibody using nuclear extract from stable cell lines expressing Flag-tagged WT or mutant histone H3 (H3.1 K115R, K122R, or K115R/K122R) shows that the anti-H3 K122Ac antibody non-specifically recognizes unmodified histone H3 and/or other modification(s) besides H3 K122Ac

Given that the function of H3 K122Ac in transcription in mammalian cells was largely defined based on its localization to active regions on chromatin [15], we investigated whether this was also the case in *Drosophila* given that we found no transcriptional defects of the markers we assayed in the *K122R* mutant clones. Using the same Abcam antibody to H3 K122Ac that was used previously during analysis of its transcriptional role [15], we found that it produced a nuclear pattern of staining in flies that was identical between wild-type and *K122R* mutant clones (Fig. 6a). Given that the flies also have the histone variant H3.3 that could potentially substitute for the canonical histones for H3 K122 Ac, we examined the specificity of the antibody directly in mammalian cells. Using cells expressing H3-YFP, we found that the ability of the H3 K122Ac antibody to recognize H3-YFP in western blots was identical for wild-type and H3 K122R mutant protein (Fig. 6b), indicating that it is non-specific in western blots, no matter how high a dilution of antibody we used (data not shown). Given that ChIP and immunofluorescence are based on recognition of the native epitope, we examined whether the H3 K122Ac antibody was specific for the native H3 K122Ac epitope. We immunoprecipitated histones with the H3 K122Ac antibody and found that it was equally as effective at immunoprecipitating Flag-tagged H3 as Flag-tagged H3 K122R histones (Fig. 6c). As such, the antibody that is commonly used to study H3 K122Ac is highly non-specific within metazoan cells, requiring the reported role of H3 K122Ac in transcription in metazoans in vivo to be revisited.

## Discussion

Here, we provide the first in vivo genetic study of the globular domain histone modifications H3 K56Ac, H3 T80p, H3 K115Ac, H3 T118p, and H3 K122Ac in a multicellular organism. We demonstrate that, with the exception of *K122R* mutations, these residues are essential for *Drosophila* development, playing roles at different times in development. H3 T118p and H3 K115Ac are needed for proper cell growth, while too much H3 K56Ac is slightly disruptive to cell growth. We also find that none of these globular domain histone modifications are required for transcription or early differentiation in imaginal discs, while the H3 K122Ac antibody used to infer its transcriptional role in metazoans is highly non-specific in vivo.

Because there is no evidence for the existence of modification of H3 T80p, K115Ac, T118p, or K122Ac in budding yeast, we used the *Drosophila* genetic system to study the functional role of this important, yet poorly studied, family of globular domain histone modifications. With the exception of H3 K115Ac and H3 K122Ac, the histone globular domain modifications that we studied all exist broadly in flies. This is the case for H3 K56Ac [9], T80p [23], and T118p [21]. No specific antibodies exist for H3 K115Ac and H3 K122Ac to determine whether they exist in flies or not. But given that H3 K122Ac exists in *pombe* [15], it is highly likely to exist in flies. Interestingly, *K122R* mutant flies appear to survive better than those rescued by WT transgenes. This result could be interpreted to mean that the modification of K122 by acetylation and/or methylation is somewhat deleterious in flies. Alternatively, the histone expression or protein stability from the H3 K122R transgenes may be in some way improved over that of WT such that the flies are more viable. Nonetheless, directly testing the role of globular domain histone PTMs by mutating the modifiable residues is especially informative because the enzymes that generate these particular modifications have numerous histone and/or non-histone targets (e.g., CBP/p300 for H3 K56Ac, H3 K115Ac, and H3 K122Ac [11] and Aurora A for H3 T118p [21]).

A surprising aspect of our study is that transcription of Wg, Ubx, and ELAV appears normal within cells that only express mutant forms of H3 K56, H3 T80, H3 K115, H3 T118, and H3 K122. This is true in both eye and wing imaginal tissues, and the presence of ELAV within mutant cells in eye discs suggests that differentiation is not generally perturbed when mutations predicted to mimic or prevent these modifications are introduced. Previous studies have indicated that both H3 K56Ac and H3 K122Ac localize to active regions of promoters and/or enhancers in mammalian cells [10, 15]. Our results suggest that K56Ac and K122Ac do not play a major role in transcription in *Drosophila* imaginal disc development. However, we cannot exclude the possibility that the replication-independent histone variant H3.3, which is encoded by two genes in *Drosophila* (H3.3A and H3.3B), could compensate for the function of our mutated canonical transgenes. While it is clear that H3.3 cannot take over the function of canonical H3.2 to permit cell proliferation, in H3.2 point mutants where cell proliferation is rescued, the corresponding *WT* residues in H3.3 could still provide partial rescue during transcription. In other words, the phenotype could be more severe if the clones were analyzed in a H3.3A/H3.3B double mutant background. These experiments should be performed in the future in order to clarify this question.

The inferred role of H3 K122Ac in transcription in mammalian cells was largely based on its immunolocalization to enhancers using a commercially available antibody [12]. Although this antibody is specific in dot blots when all acetylated peptides are present in equal amounts, the H3 K122Ac modification is relatively rare compared to N-terminal histone modifications within mammalian cells (Alan Tackett, personal communication). As such, antibody specificity has to be determined in the context of the cell due to the diverse differences in the relative abundance of different histone modifications in vivo. Accordingly, we find that the H3 K122Ac signal by immunofluorescence, western analysis, or immunoprecipitation is unchanged upon mutation of H3 K122 to a non-acetylatable residue (Fig. 6), suggesting that the H3 K122Ac antibody favors other acetylation sites in vivo. Noteworthy, our work does not rule out a role for H3 K122Ac in transcription, it just shows that it does not have a major role in transcription in *Drosophila* imaginal disc development. Indeed, we showed previously that CBP and p300 are the HATs that mediate H3 K122 acetylation [11], consistent with a role in transcription or potentially DNA repair. However, caution should be taken when interpreting experiments using the Abcam H3 K122Ac antibody, which in vivo is clearly non-specific. Similarly, much of the inferred role of H3 K56Ac in transcription in metazoan cells comes from studies with antibodies to H3 K56Ac, which shows H3 K56Ac localizing to active promoters or enhancers in mammalian cells [10, 38–44]. However, all of the commercially available H3 K56Ac antibodies are non-specific in mammalian cells, cross-reacting with other lysine residues [45] (Ohsawa and Tyler, unpublished observation). These results highlight the critical need for genetic studies to determine the true function of histone PTMs in metazoans.

A striking finding of our study is that the proliferative capacity of cells is impaired by mutation of H3 K56Q, H3 K115, H3 T118, and H3 K115/K122. The profound effect of the H3 T118 mutations on clone size and organismal viability in *Drosophila* is reminiscent of the effect of these mutations in budding yeast. H3 T118E is lethal in budding yeast, and even low-level expression disrupts transcriptional silencing [46]. Mutations of H3 K56, K115, and K122 lead to DNA damage sensitivity and loss of transcriptional silencing [6, 46]. It is possible that the transcriptional assays that we used in *Drosophila* were not sensitive enough to detect changes in silencing in cells expressing only the histone mutations. Conversely, the presence of linker histones and repressive histone modifications in *Drosophila* that are absent from yeast, may limit the ability of these mutations to destabilize nucleosomes in *Drosophila.* Importantly, while previous studies have revealed little about the function of H3 K115Ac, our results suggest a role for this modification during cell growth and/or cell division. A recent study from our laboratory suggests that H3 T118p induces a structural change within mitotic chromosomes [21]. While our analyses in *Drosophila* could not detect any overt changes in the cell cycle markers that we tested in the *H3 T118* mutant clones, their dramatically decreased ability to grow supports a crucial function for H3 T118p during mitosis. Our inability to detect cells with specific defects in the cell cycle or increased apoptosis within the mutant histone clones that are very small, such as *H3 T118I* and *T118E*, may be due to the fact that *Drosophila* has a defense system to eliminate unfit cells from developing tissues using cell competition [47].

## Conclusions

Our study provides the first tantalizing glimpse into the basic function of PTMs of the globular domain of histone H3 in vivo. Further, these animal models can be used for future functional tests during situations where the genome is stressed, such as in response to DNA damage or starvation. These types of studies would be especially enlightening in regard to H3 K56Ac, which has been suggested to function during the DNA damage response [6, 11]. Alternatively, these *Drosophila* models of globular domain H3 mutations provide an excellent tool to study the function of globular domain PTMs in stem cells, given the relative lack of understanding of how histone modifications function in those fundamentally important lineages.

## Methods

The following genotypes were used in this study:

(*Df*(*2L*)*His*^*C*^ is abbreviated *ΔHisC*).

*yw*; *ΔHisC*/*CyO*, *P*{*ActGFP*}*JMR1*; *6xHisGU*^*VK33*,*27*^*WT*/*6xHisGU*^*VK33*,*27*^*WT*

*yw*; *ΔHisC*/*CyO*, *P*{*ActGFP*}*JMR1*; *6xHisGU*^*VK33*,*27*^*H3K56R*/*6xHisGU*^*VK33*,*27*^*H3K56R*

*yw*; *ΔHisC*/*CyO*, *P*{*ActGFP*}*JMR1*; *6xHisGU*^*VK33*,*27*^*H3K56Q*/*6xHisGU*^*VK33*,*27*^*H3K56Q*

*yw*; *ΔHisC*/*CyO*, *P*{*ActGFP*}*JMR1*; *6xHisGU*^*VK33*,*27*^*H3T80A*/*6xHisGU*^*VK33*,*27*^*H3T80A*

*yw*; *ΔHisC*/*CyO*, *P*{*ActGFP*}*JMR1*; *6xHisGU*^*VK33*,*27*^*H3T80E*/*6xHisGU*^*VK33*,*27*^*H3T80E*

*yw*; *ΔHisC*/*CyO*, *P*{*ActGFP*}*JMR1*; *6xHisGU*^*VK33*,*27*^*H3K115R*/*6xHisGU*^*VK33*,*27*^*H3K115R*

*yw*; *ΔHisC*/*CyO*, *P*{*ActGFP*}*JMR1*; *6xHisGU*^*VK33*,*27*^*H3K115Q*/*6xHisGU*^*VK33*,*27*^*H3K115Q*

*yw*; *ΔHisC*/*CyO*, *P*{*ActGFP*}*JMR1*; *6xHisGU*^*VK33*,*27*^*H3T118A*/*6xHisGU*^*VK33*,*27*^*H3T118A*

*yw*; *ΔHisC*/*CyO*, *P*{*ActGFP*}*JMR1*; *6xHisGU*^*VK33*,*27*^*H3T118E*/*6xHisGU*^*VK33*,*27*^*H3T118E*

*yw*; *ΔHisC*/*CyO*, *P*{*ActGFP*}*JMR1*; *6xHisGU*^*VK33*,*27*^*H3T118I*/*6xHisGU*^*VK33*,*27*^*H3T118I*

*yw*; *ΔHisC*/*CyO*, *P*{*ActGFP*}*JMR1*; *6xHisGU*^*VK33*,*27*^*H3K122R*/*6xHisGU*^*VK33*,*27*^*H3K122R*

*yw*; *ΔHisC*/*CyO*, *P*{*ActGFP*}*JMR1*; *6xHisGU*^*VK33*,*27*^*H3K122Q*/*6xHisGU*^*VK33*,*27*^*H3K122Q*

*yw*; *ΔHisC*/*CyO*, *P*{*ActGFP*}*JMR1*; *6xHisGU*^*VK33*,*27*^*H3K115R*–*K122R*/*6xHisGU*^*VK33*,*27*^*H3K115R*-*K122R*

*yw*; *ΔHisC*/*CyO*, *P*{*ActGFP*}*JMR1*; *6xHisGU*^*VK33*,*27*^*H3K115Q*–*K122Q*/*6xHisGU*^*VK33*,*27*^*H3K115Q*-*K122Q*

*yw*; *ΔHisC FRT40A*/*CyO*, *P*{*ActGFP*}*JMR1* (*or CyO*, *P*{*MHC*-*RFP*})

*w*[*1118*]; *P*{*w*[+*mC*] = *piM*}*36F P*{*ry*[+*t7.2*] = *neoFRT*}*40A* (*Bloomington stock #1818*)

*yw*

*GMR*-*hid*

*yw*; *ΔHisC FRT40A*; *6xHisGU*^*VK33*,*27*^*WT*/*SM5^TM6B*

*yw*; *ΔHisC FRT40A*; *6xHisGU*^*VK33*,*27*^*H3K56R*/*SM5^TM6B*

*yw*; *ΔHisC FRT40A*; *6xHisGU*^*VK33*,*27*^*H3K56Q*/*SM5^TM6B*

*yw*; *ΔHisC FRT40A*; *6xHisGU*^*VK33*,*27*^*H3T80A*/*SM5^TM6B*

*yw*; *ΔHisC FRT40A*; *6xHisGU*^*VK33*,*27*^*H3T80E*/*SM5^TM6B*

*yw*; *ΔHisC FRT40A*; *6xHisGU*^*VK33*,*27*^*H3K115R*/*SM5^TM6B*

*yw*; *ΔHisC FRT40A*; *6xHisGU*^*VK33*,*27*^*H3K115Q*/*SM5^TM6B*

*yw*; *ΔHisC FRT40A*; *6xHisGU*^*VK33*,*27*^*H3T118A*/*SM5^TM6B*

*yw*; *ΔHisC FRT40A*; *6xHisGU*^*VK33*,*27*^*H3T118E*/*SM5^TM6B*

*yw*; *ΔHisC FRT40A*; *6xHisGU*^*VK33*,*27*^*H3T118I*/*SM5^TM6B*

*yw*; *ΔHisC FRT40A*; *6xHisGU*^*VK33*,*27*^*H3K122R*/*SM5^TM6B*

*yw*; *ΔHisC FRT40A*; *6xHisGU*^*VK33*,*27*^*H3K122Q*/*SM5^TM6B*

*yw*; *ΔHisC FRT40A*; *6xHisGU*^*VK33*,*27*^*H3K115R*-*K122R*//*SM5^TM6B*

*yw*; *ΔHisC FRT40A*; *6xHisGU*^*VK33*,*27*^*H3K115Q*-*K122Q*/*SM5^TM6B*

(*X* = *ubx*, *ey*, or *hs122* in the genotypes below; specific flippases can be found in the figure legends)

*yw X*-*Flp*; *ubiGFP FRT40A*; *6xHisGU*^*VK33*,*27*^*WT*/*6xHisGU*^*VK33*,*27*^*WT*

*yw X*-*Flp*; *ubiGFP FRT40A*; *6xHisGU*^*VK33*,*27*^*H3K56R*/*6xHisGU*^*VK33*,*27*^*H3K56R*

*yw X*-*Flp*; *ubiGFP FRT40A*; *6xHisGU*^*VK33*,*27*^*H3K56Q*/*6xHisGU*^*VK33*,*27*^*H3K56Q*

*yw X*-*Flp*; *ubiGFP FRT40A*; *6xHisGU*^*VK33*,*27*^*H3T80A*/*6xHisGU*^*VK33*,*27*^*H3T80A*

*yw X*-*Flp*; *ubiGFP FRT40A*; *6xHisGU*^*VK33*,*27*^*H3T80E*/*6xHisGU*^*VK33*,*27*^*H3T80E*

*yw X*-*Flp*; *ubiGFP FRT40A*; *6xHisGU*^*VK33*,*27*^*H3K115R*/*6xHisGU*^*VK33*,*27*^*H3K115R*

*yw X*-*Flp*; *ubiGFP FRT40A*; *6xHisGU*^*VK33*,*27*^*H3K115Q*/*6xHisGU*^*VK33*,*27*^*H3K115Q*

*yw X*-*Flp*; *ubiGFP FRT40A*; *6xHisGU*^*VK33*,*27*^*H3T118A*/*6xHisGU*^*VK33*,*27*^*H3T118A*

*yw X*-*Flp*; *ubiGFP FRT40A*; *6xHisGU*^*VK33*,*27*^*H3T118E*/*6xHisGU*^*VK33*,*27*^*H3T118E*

*yw X*-*Flp*; *ubiGFP FRT40A*; *6xHisGU*^*VK33*,*27*^*H3T118I*/*6xHisGU*^*VK33*,*27*^*H3T118I*

*yw X*-*Flp*; *ubiGFP FRT40A*; *6xHisGU*^*VK33*,*27*^*H3K122R*/*6xHisGU*^*VK33*,*27*^*H3K122R*

*yw X*-*Flp*; *ubiGFP FRT40A*; *6xHisGU*^*VK33*,*27*^*H3K122Q*/*6xHisGU*^*VK33*,*27*^*H3K122Q*

*yw X*-*Flp*; *ubiGFP FRT40A*; *6xHisGU*^*VK33*,*27*^*H3K115R*-*K122R*/*6xHisGU*^*VK33*,*27*^*H3K115R*-*K122R*

*yw X*-*Flp*; *ubiGFP FRT40A*; *6xHisGU*^*VK33*,*27*^*H3K115Q*-*K122Q*/*6xHisGU*^*VK33*,*27*^*H3K115Q*-*K122Q*

### Generation of histone mutant flies

We constructed *6xHisGU*^*VK33*,*27*^*WT* and the *6xHisGU*^*VK33*,*27*^*H3 mutant* chromosomes essentially as previously described [26] with the following changes:

ΦC31attB3xHisGU.H3K56R, ΦC31attB3xHisGU.H3K56Q, ΦC31attB3xHisGU.H3T80A, and ΦC31attB3xHisGU.H3T08E plasmids were generated by replacing the XcmI/SacI fragment in pENTR221-HisGU with a synthetic fragment (Integrated DNA Technologies, Inc., Coralville IA, USA) containing an AAG into CGC codon exchange leading to the H3 K56R mutation, an AAG into CAG codon exchange resulting in H3 K56Q, an ACG into GCC codon exchange resulting in H3T80A, and an ACG into GAG codon exchange resulting in H3T80E. ΦC31attB3xHisGU.H3K115R, ΦC31attB3xHisGU.H3K115Q, ΦC31attB3xHisGU.H3T118A, ΦC31attB3xHisGU.H3T118E, ΦC31attB3xHisGU.H3T118I, ΦC31attB3xHisGU.H3K122R, ΦC31attB3xHisGU.H3K122Q, ΦC31attB3xHisGU.H3K115R/K122R, and ΦC31attB3xHisGU.H3K115Q/K122Q plasmids were generated by replacing the EcoRI/SacI fragment in pENTR221-HisGU with a synthetic fragment (Integrated DNA Technologies, Inc., Coralville, IA, USA) containing an AAG into CGC codon exchange leading to the H3K115R mutation, an AAG into CAG codon exchange resulting in H3K115Q, an ACC into GCC codon exchange resulting in H3 T118A, an ACC into GAG codon exchange resulting in H3 T118E, an ACC into AUC codon exchange resulting in H3 T118I mutation, an AAA into CGC codon resulting in H3 K122R, an AAA into CAG codon exchange resulting in H3 K122Q, and the same codon exchanges combined creating the various K115/K122 double mutants. All plasmids were sequenced to verify the presence of the mutations. We utilized ΦC31-mediated transgenesis to integrate the ΦC31attB3xHisGU.H3mutant constructs as well as ΦC31attB3xHisGU.WT site specifically into the *Drosophila* genome using the landing sites *VK27* (on 3R) and *VK33* (on 3L) [48]. Homozygous viable insertions from each site, which displayed orange to dark orange eyes, were recombined to generate *6xHisGU*^*VK33*,*27*^*WT* and *6xHisGU*^*VK3*3,*27*^*H3mutant* chromosomes, which displayed red eyes, and crossed into the *ΔHisC* mutant background [26]. Five independent *6xHisGU*^*VK33*,*27*^ recombinants were tested for each mutant for their ability to rescue *ΔHisC* homozygous mutants, and two of those recombinants were tested in clonal analysis experiments to ensure consistency.

### Lethal phase analysis

For lethal phase experiments *ΔHisC* was kept heterozygous over *CyO*, *P*{*ActGFP*}*JMR1* to identify mutant embryos or larvae lacking green fluorescent protein expression, and the viability of *12xHisGU* transgene containing mutant and wild-type animals was assessed (>100 animals per genotype). Wild-type controls were either non-mutant sibling embryos (internal control) or embryos which contain *12xHisGU* (WT control).

### Quantification of clone area in wing discs

Mutant clone area for multiple wing discs of each genotype was quantified by analyzing a 40 × image of the disc proper around the wing pouch region using the thresholding function in the Image J software. Hue and brightness were adjusted such that only mutant clones (which are DAPI+ and GFP−) were selected using the magic wand tool, and then the measure function was used to obtain the total mutant clone area in pixels. Mutant clone areas from each genotype were compiled and then compared to wild type using paired *t* tests. Sample sizes were as follows (genotype, *n* = 72 h, *n* = 96 h): *ΔHisC FRT40A*, *n* = 10, *n* = 10; *FRT40A*, *n* = 21, *n* = 20; *WT*, *n* = 20, *n* = 15; *K56R*, *n* = 15, *n* = 10; *K56Q*, *n* = 12, *n* = 6; *T80A*, *n* = 14, *n* = 12; *T80E*, *n* = 14, *n* = 11; *K115R*, *n* = 23, *n* = 22; *K115Q*, *n* = 21, *n* = 25; *T118A*, *n* = 14, *n* = 25; *T118E*, *n* = 7, *n* = 20; *T118I*, *n* = 10, *n* = 13; *K122R*, *n* = 10, *n* = 12; *K122Q*, *n* = 13, *n* = 10; *K115R*/*K122R*, *n* = 23, *n* = 12; *K115Q*/*K122Q*, *n* = 6, *n* = 12.

### Chi-squared analysis

Progeny from *yw*; *ΔHisC FRT40A*; *6xHisGU*^*VK3*3,*27*^*WT or H3mutant*/*SM5*^*TM6B x yw*; *ΔHisC FRT40A*; *6xHisGU*^*VK33*,*27*^*WT or H3mutant/SM5^TM6B* were scored from at least 10 vials per genotype to assess the ability of *12xHisGU*^*VK33*,*27*^*WT or H3mutant* transgenes to rescue the homozygous *ΔHisC* mutant to adulthood. Notably, *ΔHisC*; *12xHisGU*^*VK3*3,*27*^*WT* animals were able to survive to adulthood with no visible phenotypes, but we were unable to establish a stock of the animals, suggesting a possible fertility problem. Also, some pupal lethality was observed in the non-TM6B progeny. Therefore, viability was quantified by counting the number of empty non-TM6B pupae (*ΔHisC*; *12xHisGU*^*VK33*,*27*^*WT or H3 mutant*) versus the number of TM6B and non-TM6B pupae that had pharate adults within them (*ΔHisC FRT40A*; *6xHisGU*^*VK33*,*27*^*WT or H3mutant*/*SM5*^*TM6B* and non-viable *ΔHisC*; *12xHisGU*^*VK33*,*27*^*WT* or *H3mutant*) in fully enclosed vials of flies. Normalized expected values for each genotype were compared to observed values using a Chi-squared test.

### Antibody staining

Dissections and staining of wing and eye discs were performed according to standard protocols. Primary antibodies used in this study were Wg (1:10, DSHB), Ubx (1:10, DSHB), H3 S10p (1:500–1:1000 Abcam), H3 K122Ac (1:250–1:500, Abcam #ab33309), and ELAV (1:50, DSHB). BrdU and TUNEL assays were performed as previously described [49–51]. Secondary antibodies used were Alexa488 or Cy3 (1:600, Jackson Immunoresearch). More than 15 discs were analyzed for each marker assayed. Images were obtained using a FV1000 Olympus confocal, and all images represent Z-stacks of the disc proper portion of the imaginal disc.

### Western blots

Plasmids expressing YFP-tagged mutant H3.1 (K115R, K122R, T118E or K115RK122R) were generated by site-directed mutagenesis on the pcDNA5-wild-type H3.1-YFP using QuickChange Site-Directed Mutagenesis Kit (Agilent Technologies). HEK293T cells were transiently transfected with YFP-tagged WT or mutant histone H3.1 expression plasmid (or pcDNA5 empty plasmid as a control). Cells were harvested 24 h posttransfection and processed for histone acid extraction. Acid-extracted histones were separated by SDS-PAGE, probed with the anti-H3 K122Ac antibody (Rabbit pAb: Abcam 33309) and anti-GFP antibody (Mouse). The secondary antibody IRDye^®^ 680RD Goat anti-Rabbit IgG (H + L) multiplexed with the IRDye^®^ 800CW Goat anti-Mouse IgG (H + L) was used for the two-color detection method by the Odyssey LI-COR imaging system.

### Immunoprecipitation

Plasmids expressing Flag-tagged mutant H3.1 (K115R, K122R or K115RK122R) were generated by site-directed mutagenesis on the pcDNA5/FRT-wild-type H3.1-FLAG using QuickChange Site-Directed Mutagenesis Kit (Agilent Technologies). Flp-In™-293 host cells were co-transfected with plasmid pcDNA5/FRT-H3.1-FLAG (either WT or mutant H3.1, i.e., H3.1 K115R, K122R, or K115RK122R) and Flp recombinase expression plasmid pOG44, to generate cell lines that stably express Flag-tagged WT or mutant histone H3.1. The stable cells lines were selected based on their resistance to hygromycin, sensitivity to zeocin, and expression of Flag-tagged histone H3. Stable Flp-In™-293 cells were harvested and processed for nuclear extract, as previously described [23]. Twenty microliters of Dynabeads Protein A and 1 μg of the Abcam anti-H3 K122Ac antibody (Rabbit pAb: Abcam 33309) were used for each IP sample which contained 300 μg of total proteins. The eluted supernatants were separated by SDS-PAGE, probed with the anti-H3 antibody (Rabbit pAb: Abcam 1791) and anti-Flag antibody (Mouse mAb: Sigma 3165). The secondary antibody IRDye^®^ 680RD Goat anti-Rabbit IgG (H + L) multiplexed with the IRDye^®^ 800CW Goat anti-Mouse IgG (H + L) was used for the two-color detection method by the Odyssey LI-COR imaging system.

## Additional files

10.1186/s13072-016-0059-3 BrdU is incorporated within cells that have mutations which prevent/mimic globular domain histone H3 modifications. A) *yw* control wing imaginal discs. Merged images show the nuclear marker DAPI in blue and BrdU in magenta. Grayscale images are the individual BrdU channels. B-P) Wing imaginal discs with GFP negative mutant clones generated using *Ubx*-*FLP*. Merged images show the nuclear marker DAPI in blue, BrdU in magenta, and GFP^+^ and GFP^-^ regions demarcate histone wild type cells and histone mutant cells, respectively. Grayscale images are the individual BrdU channels. Within each mutant, we looked at GFP^-^ clones within the zone of non-proliferation of the wing disc (arrow in B) to determine if there was a consistent increase of BrdU incorporation, which might indicate an increase of DNA synthesis. Conversely, we looked at GFP^-^ clones outside of the zone of non-proliferation to determine if there was a consistent decrease of BrdU incorporation compared to their neighboring GFP^+^ control cells.
10.1186/s13072-016-0059-3 H3S10p expression is not changed within cells that have mutations which prevent/mimic globular domain histone H3 modifications. A-N) Wing imaginal discs with GFP negative mutant clones generated using *Ubx*-*FLP*. Merged images show the nuclear marker DAPI in blue, H3S10p in magenta, and GFP^+^ and GFP^-^ regions demarcate histone wild type cells and histone mutant cells, respectively. Grayscale images are the individual H3S10p channels. Within each mutant, we looked at GFP^-^ clones within the zone of non-proliferation of the wing disc (see Sup. Fig. 4B) to determine if there was a consistent increase of H3S10p incorporation, which might indicate an increase of mitotic cells. Conversely, we looked at GFP^-^ clones outside of the zone of non-proliferation to determine if there was a consistent decrease of H3S10p incorporation compared to their neighboring GFP^+^ control cells.
10.1186/s13072-016-0059-3 TUNEL assay within H3 mutant mosaic discs. A and B) *GMR*-*hid* and *yw* control eye and wing imaginal discs. Merged images show the nuclear marker DAPI in blue and TUNEL in magenta. Grayscale images are the individual TUNEL channels. The pattern of TUNEL in *GMR*-*hid*, a fly mutant that expresses the cell death gene *hid* in the posterior of the developing eye, eye discs was used as a positive control (arrow in A). Note that the edge of the wing disc is often TUNEL positive in the control, as well as the mutants. This is a consequence of background staining of TUNEL in the peripodium which wraps around the edge of the disc. All images are Z-stacks of clones in the disc proper, but some of the peripodial epithelium that is wrapped around the disc appears in the Z-stack. C-P) Wing imaginal discs with GFP negative mutant clones generated using *Ubx*-*FLP*. Merged images show the nuclear marker DAPI in blue, TUNEL in magenta, and GFP^+^ and GFP^-^ regions demarcate histone wild type cells and histone mutant cells, respectively. Grayscale images are the individual TUNEL channels. Within each mutant genotype, we looked at GFP^-^ clones outside the edges of the disc to determine if there was a consistent increase of TUNEL compared to their neighboring GFP^+^ control cells.
10.1186/s13072-016-0059-3 Markers to developmental signaling pathways are normal within cells that have mutations which prevent/mimic globular domain histone H3 modifications. A-N) Wing imaginal discs with GFP negative mutant clones generated using *Ubx*-*FLP*. Merged images show the nuclear marker DAPI in blue, Wg in magenta, and GFP^+^ and GFP^-^ regions demarcate histone wild type cells and histone mutant cells, respectively. Grayscale images are the individual Wg channels.
10.1186/s13072-016-0059-3 Developmental signaling pathways are not derepressed within cells that have mutations which prevent/mimic globular domain histone H3 modifications. A-N) Wing imaginal discs with GFP negative mutant clones generated using *Ubx*-*FLP*. Merged images show the nuclear marker DAPI in blue, Ubx in magenta, and GFP^+^ and GFP^-^ regions demarcate histone wild type cells and histone mutant cells, respectively. Grayscale images are the individual Ubx channels. Note that Ubx is expressed in the peripodial membrane of wing discs, tracheal cells that are accidently attached to the wing discs, as well as in leg discs, allowing for a positive control for the staining.
10.1186/s13072-016-0059-3 Table of phenotypes observed in adult eyes of *yw eyFLP; ubiGFP FRT40A/ΔHisC FRT40A; 12xHisGUH3 mutants.* It is important to keep in mind that rapid death of mutant cells after clone induction (e.g. like *∆HisC* cells) is normally less of a problem for the animal because the surrounding wild-type cells compensate and imaginal disc tissues form and differentiate normally. Rather, it is in the case where cells survive and proliferate but are not able to execute their normal developmental program, where larvae generally tend to get sick and the differentiated tissues in adults show defects. Because of the presence of white + and yellow + marker genes in the histone transgene cassette inserts, it is not possible to generate yellow- or white- marked ∆HisC clones in adult tissues. Therefore, the genotype of the cells that form the differentiated structures (i.e. the eye) is not known and it is not known whether the rough eye phenotype is caused by abnormally differentiated mutant cells or by death of the mutant cells and inability of the surrounding healthy tissue to compensate.

## Abbreviations

PTM: post-translational modification
Ac: acetylation
p: phosphorylation
me: methylation
ub: ubiquitination
