# Supplementary material for: Mutations that prevent or mimic persistent post-translational modifications of the histone H3 globular domain cause lethality and growth defects in Drosophila
Source: Epigenetics Chromatin. 2016 Feb 29;9:9. doi: 10.1186/s13072-016-0059-3 (PMC4772521; doi:10.1186/s13072-016-0059-3)
Supplement: Supplementary file 1 — 10.1186/s13072-016-0059-3 BrdU is incorporated within cells that have mutations which prevent/mimic globular domain histone H3 modifications. A) yw control wing imaginal discs. Merged images show the nuclear marker DAPI in blue and BrdU in magenta. Grayscale images are the individual BrdU channels. B-P) Wing imaginal discs with GFP negative mutant clones generated using Ubx-FLP. Merged images show the nuclear marker DAPI in blue, BrdU in magenta, and GFP+ and GFP- regions demarcate histone wild type cells and histone mutant cells, respectively. Grayscale images are the individual BrdU channels. Within each mutant, we looked at GFP- clones within the zone of non-proliferation of the wing disc (arrow in B) to determine if there was a consistent increase of BrdU incorporation, which might indicate an increase of DNA synthesis. Conversely, we looked at GFP- clones outside of the zone of non-proliferation to determine if there was a consistent decrease of BrdU incorporation compared to their neighboring GFP+ control cells. [file 13072_2016_59_MOESM1_ESM.pptx]

## Slide 1
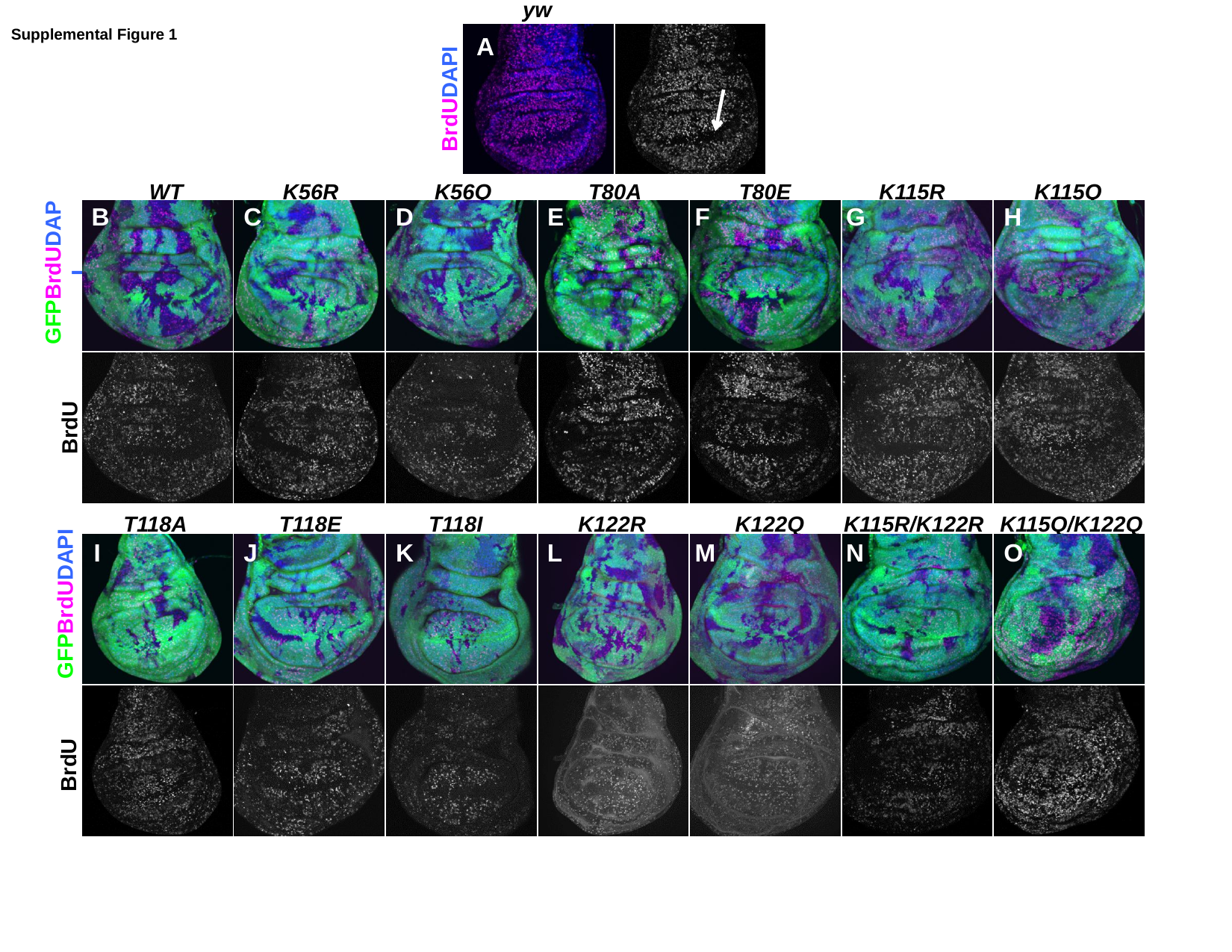

yw
A
BrdUDAPI
WT
K56R
K56Q
T80A
T80E
K115R
K115Q
B
C
D
E
F
G
H
GFPBrdUDAPI
BrdU
T118I
T118A
T118E
K122R
K122Q
K115R/K122R
K115Q/K122Q
I
J
K
L
M
N
O
GFPBrdUDAPI
BrdU
Supplemental Figure 1
