# Supplementary material for: Mutations that prevent or mimic persistent post-translational modifications of the histone H3 globular domain cause lethality and growth defects in Drosophila
Source: Epigenetics Chromatin. 2016 Feb 29;9:9. doi: 10.1186/s13072-016-0059-3 (PMC4772521; doi:10.1186/s13072-016-0059-3)
Supplement: Supplementary file 2 — 10.1186/s13072-016-0059-3 H3S10p expression is not changed within cells that have mutations which prevent/mimic globular domain histone H3 modifications. A-N) Wing imaginal discs with GFP negative mutant clones generated using Ubx-FLP. Merged images show the nuclear marker DAPI in blue, H3S10p in magenta, and GFP+ and GFP- regions demarcate histone wild type cells and histone mutant cells, respectively. Grayscale images are the individual H3S10p channels. Within each mutant, we looked at GFP- clones within the zone of non-proliferation of the wing disc (see Sup. Fig. 4B) to determine if there was a consistent increase of H3S10p incorporation, which might indicate an increase of mitotic cells. Conversely, we looked at GFP- clones outside of the zone of non-proliferation to determine if there was a consistent decrease of H3S10p incorporation compared to their neighboring GFP+ control cells. [file 13072_2016_59_MOESM2_ESM.pptx]

## Slide 1
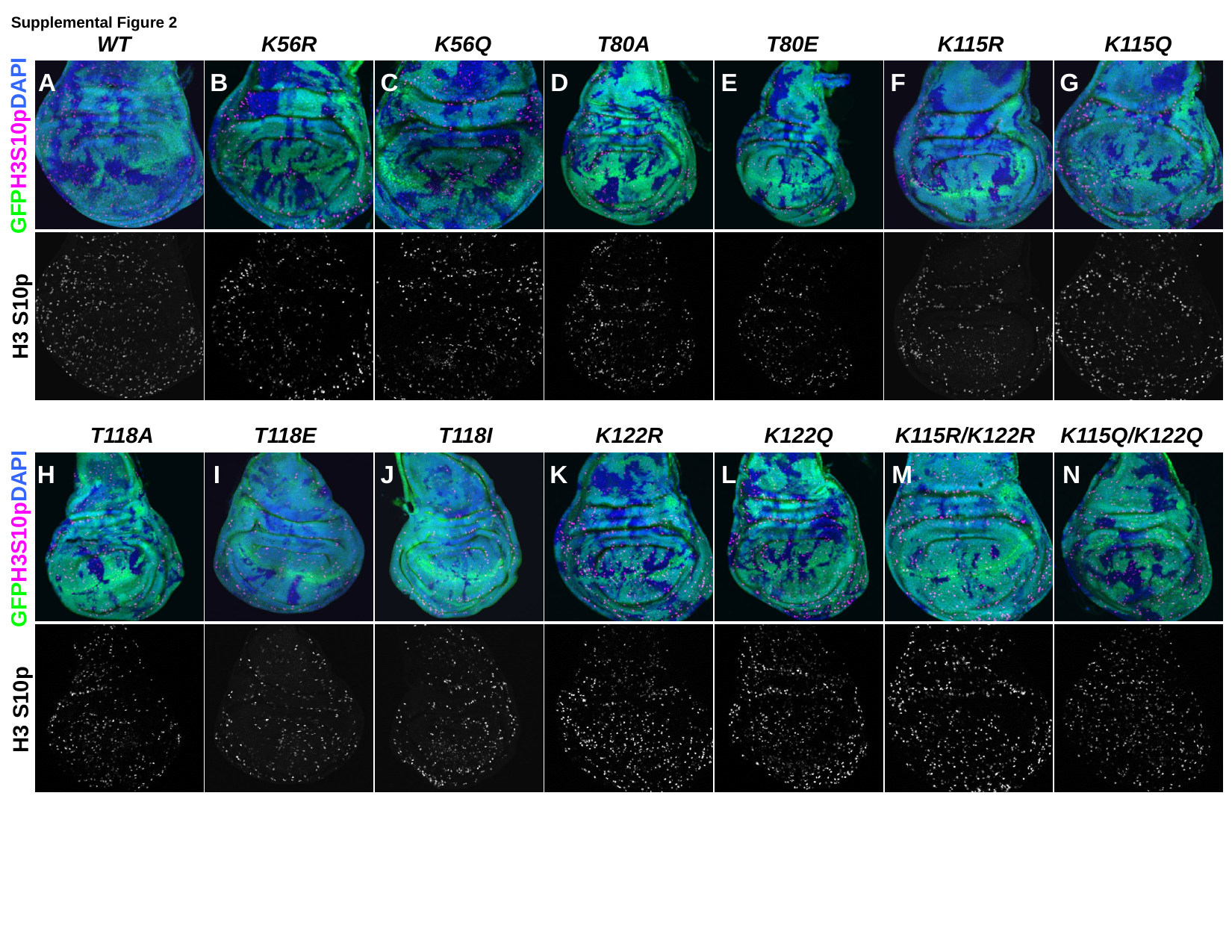

Supplemental Figure 2
WT
K56R
K56Q
T80A
T80E
K115R
K115Q
A
B
C
D
E
F
G
GFPH3S10pDAPI
H3 S10p
T118A
T118E
T118I
K122R
K122Q
K115R/K122R
K115Q/K122Q
H
I
J
K
L
M
N
GFPH3S10pDAPI
H3 S10p
