# Supplementary material for: Mutations that prevent or mimic persistent post-translational modifications of the histone H3 globular domain cause lethality and growth defects in Drosophila
Source: Epigenetics Chromatin. 2016 Feb 29;9:9. doi: 10.1186/s13072-016-0059-3 (PMC4772521; doi:10.1186/s13072-016-0059-3)
Supplement: Supplementary file 3 — 10.1186/s13072-016-0059-3 TUNEL assay within H3 mutant mosaic discs. A and B) GMR-hid and yw control eye and wing imaginal discs. Merged images show the nuclear marker DAPI in blue and TUNEL in magenta. Grayscale images are the individual TUNEL channels. The pattern of TUNEL in GMR-hid, a fly mutant that expresses the cell death gene hid in the posterior of the developing eye, eye discs was used as a positive control (arrow in A). Note that the edge of the wing disc is often TUNEL positive in the control, as well as the mutants. This is a consequence of background staining of TUNEL in the peripodium which wraps around the edge of the disc. All images are Z-stacks of clones in the disc proper, but some of the peripodial epithelium that is wrapped around the disc appears in the Z-stack. C-P) Wing imaginal discs with GFP negative mutant clones generated using Ubx-FLP. Merged images show the nuclear marker DAPI in blue, TUNEL in magenta, and GFP+ and GFP- regions demarcate histone wild type cells and histone mutant cells, respectively. Grayscale images are the individual TUNEL channels. Within each mutant genotype, we looked at GFP- clones outside the edges of the disc to determine if there was a consistent increase of TUNEL compared to their neighboring GFP+ control cells. [file 13072_2016_59_MOESM3_ESM.pptx]

## Slide 1
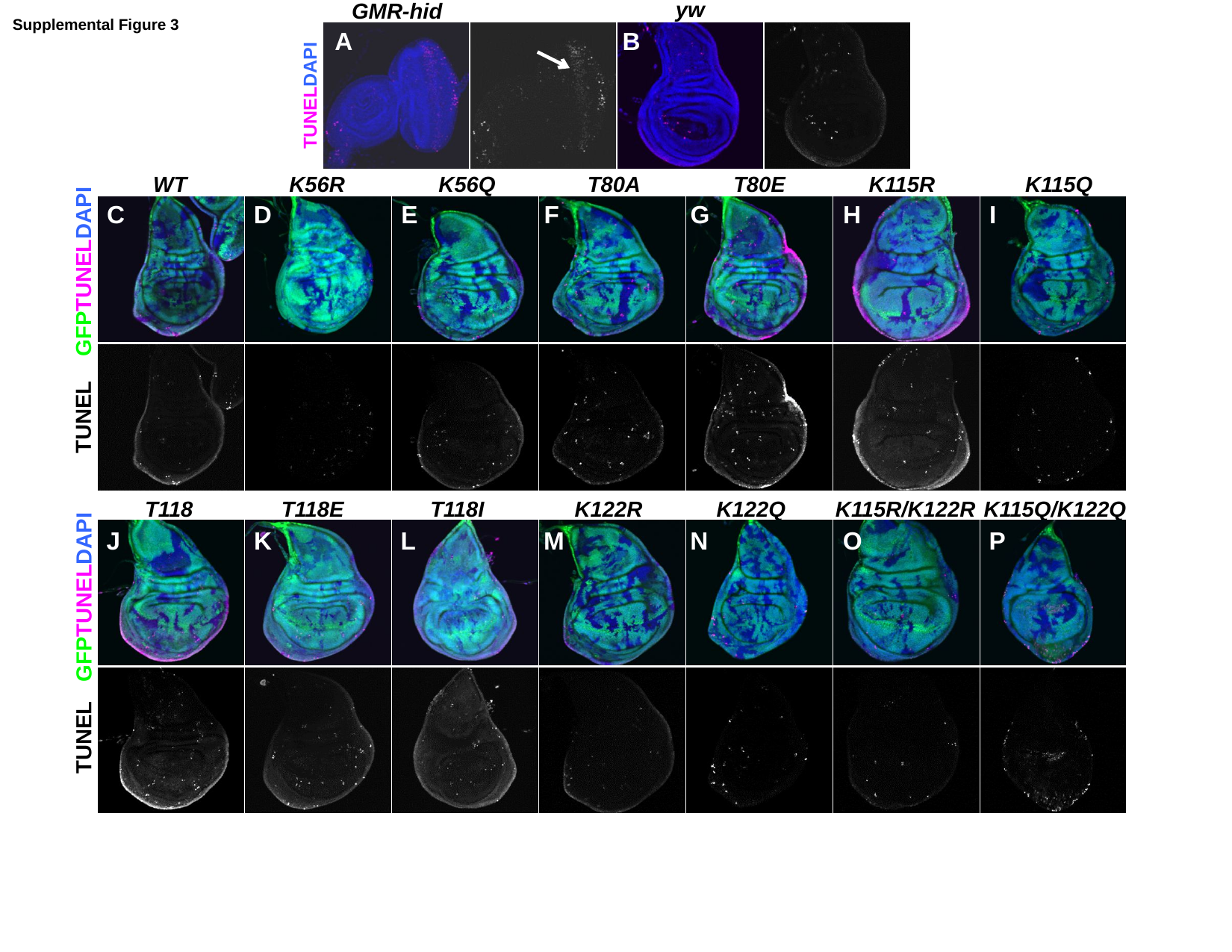

yw
GMR-hid
A
B
TUNELDAPI
WT
K56R
K56Q
T80A
T80E
K115R
K115Q
C
D
E
F
G
H
I
GFPTUNELDAPI
TUNEL
T118A
T118E
T118I
K122R
K122Q
K115R/K122R
K115Q/K122Q
J
K
L
M
N
O
P
GFPTUNELDAPI
TUNEL
Supplemental Figure 3
