# Supplementary material for: Mutations that prevent or mimic persistent post-translational modifications of the histone H3 globular domain cause lethality and growth defects in Drosophila
Source: Epigenetics Chromatin. 2016 Feb 29;9:9. doi: 10.1186/s13072-016-0059-3 (PMC4772521; doi:10.1186/s13072-016-0059-3)
Supplement: Supplementary file 5 — 10.1186/s13072-016-0059-3 Developmental signaling pathways are not derepressed within cells that have mutations which prevent/mimic globular domain histone H3 modifications. A-N) Wing imaginal discs with GFP negative mutant clones generated using Ubx-FLP. Merged images show the nuclear marker DAPI in blue, Ubx in magenta, and GFP+ and GFP- regions demarcate histone wild type cells and histone mutant cells, respectively. Grayscale images are the individual Ubx channels. Note that Ubx is expressed in the peripodial membrane of wing discs, tracheal cells that are accidently attached to the wing discs, as well as in leg discs, allowing for a positive control for the staining. [file 13072_2016_59_MOESM5_ESM.pptx]

## Slide 1
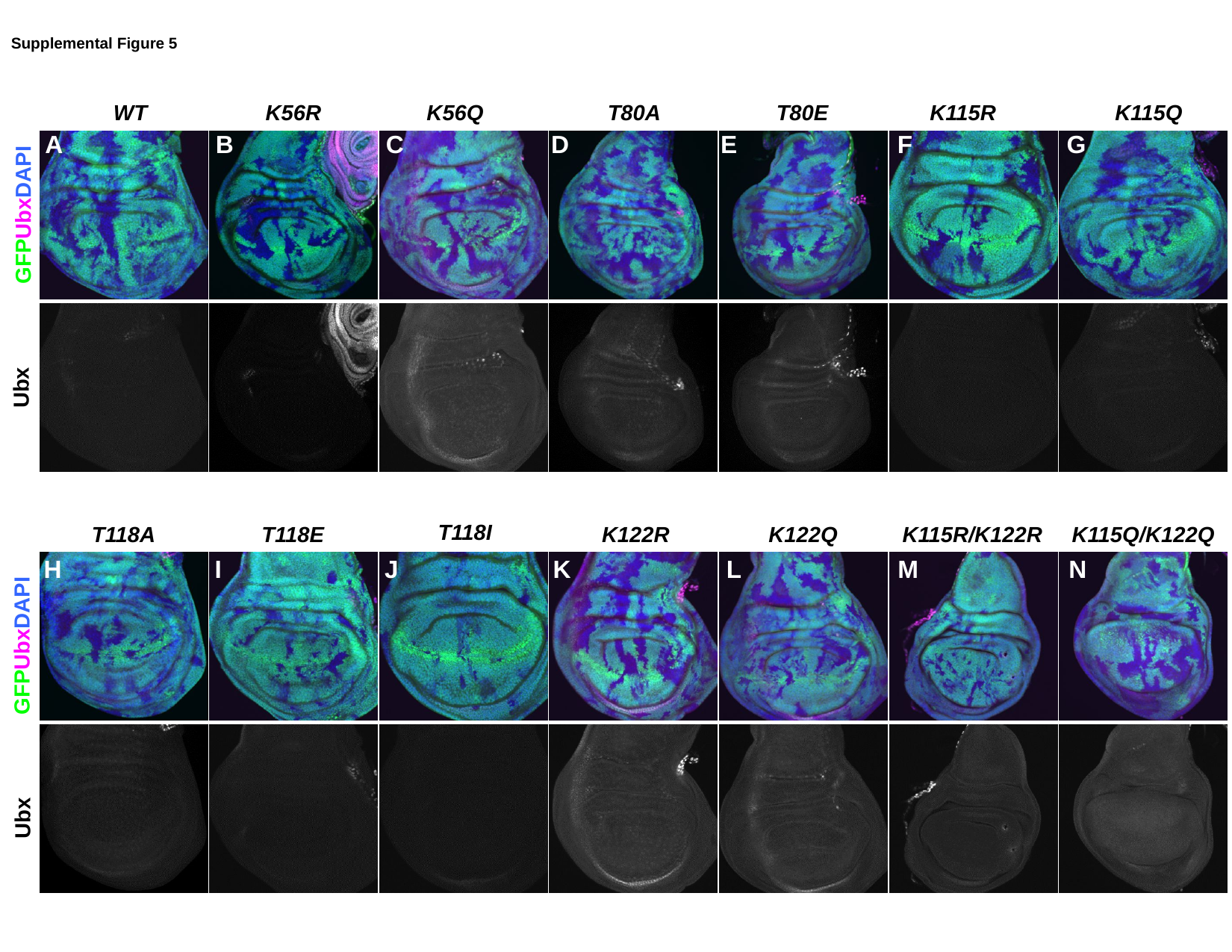

Supplemental Figure 5
WT
K56R
K56Q
T80A
T80E
K115R
K115Q
A
B
C
D
E
F
G
GFPUbxDAPI
Ubx
T118I
T118A
T118E
K122R
K122Q
K115R/K122R
K115Q/K122Q
H
I
J
K
L
M
N
GFPUbxDAPI
Ubx
