# Supplementary material for: Mutations that prevent or mimic persistent post-translational modifications of the histone H3 globular domain cause lethality and growth defects in Drosophila
Source: Epigenetics Chromatin. 2016 Feb 29;9:9. doi: 10.1186/s13072-016-0059-3 (PMC4772521; doi:10.1186/s13072-016-0059-3)
Supplement: Supplementary file 6 — 10.1186/s13072-016-0059-3 Table of phenotypes observed in adult eyes of yw eyFLP; ubiGFP FRT40A/ΔHisC FRT40A; 12xHisGUH3 mutants. It is important to keep in mind that rapid death of mutant cells after clone induction (e.g. like ∆HisC cells) is normally less of a problem for the animal because the surrounding wild-type cells compensate and imaginal disc tissues form and differentiate normally. Rather, it is in the case where cells survive and proliferate but are not able to execute their normal developmental program, where larvae generally tend to get sick and the differentiated tissues in adults show defects. Because of the presence of white + and yellow + marker genes in the histone transgene cassette inserts, it is not possible to generate yellow- or white- marked ∆HisC clones in adult tissues. Therefore, the genotype of the cells that form the differentiated structures (i.e. the eye) is not known and it is not known whether the rough eye phenotype is caused by abnormally differentiated mutant cells or by death of the mutant cells and inability of the surrounding healthy tissue to compensate. [file 13072_2016_59_MOESM6_ESM.pptx]

## Slide 1
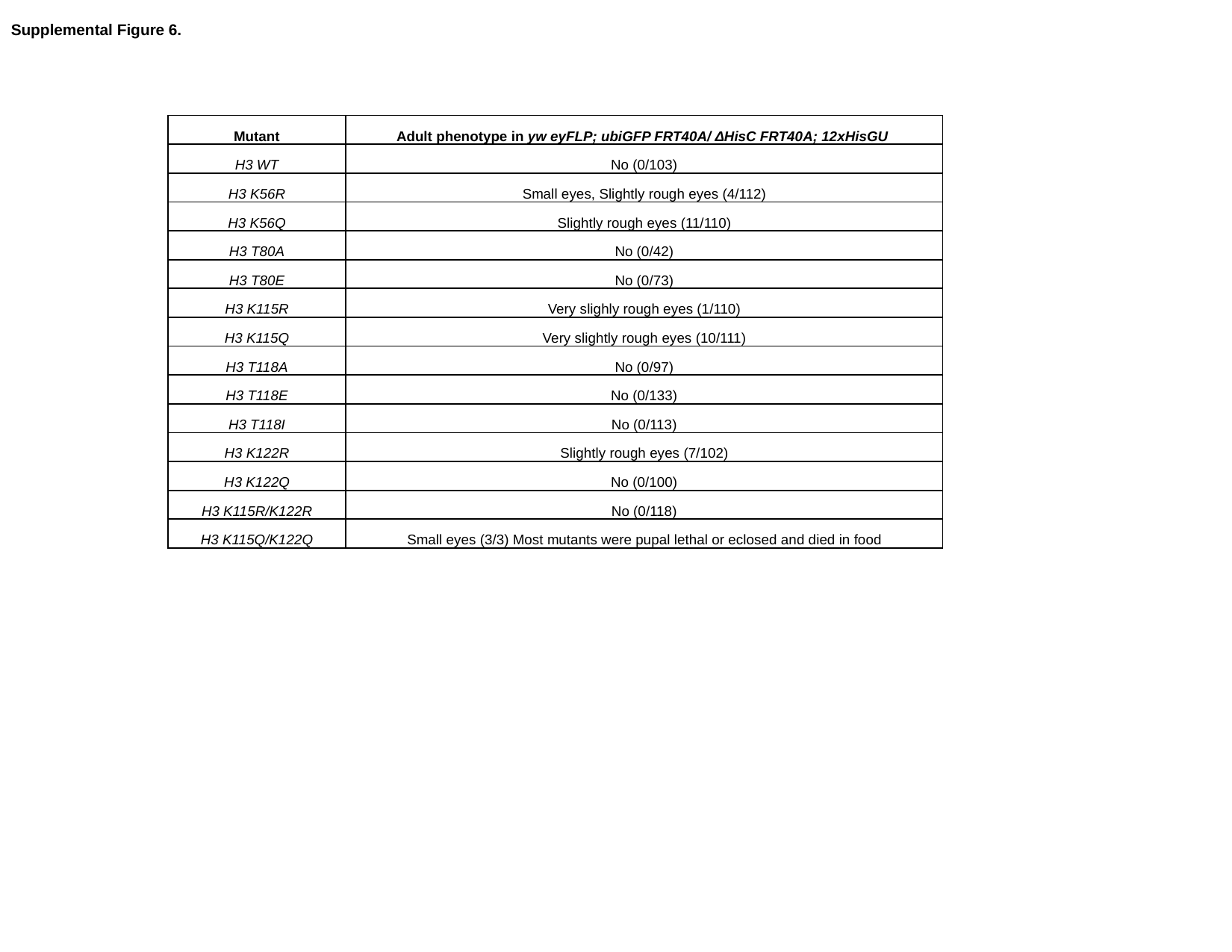

Supplemental Figure 6.
| Mutant | Adult phenotype in yw eyFLP; ubiGFP FRT40A/ ΔHisC FRT40A; 12xHisGU |
| --- | --- |
| H3 WT | No (0/103) |
| H3 K56R | Small eyes, Slightly rough eyes (4/112) |
| H3 K56Q | Slightly rough eyes (11/110) |
| H3 T80A | No (0/42) |
| H3 T80E | No (0/73) |
| H3 K115R | Very slighly rough eyes (1/110) |
| H3 K115Q | Very slightly rough eyes (10/111) |
| H3 T118A | No (0/97) |
| H3 T118E | No (0/133) |
| H3 T118I | No (0/113) |
| H3 K122R | Slightly rough eyes (7/102) |
| H3 K122Q | No (0/100) |
| H3 K115R/K122R | No (0/118) |
| H3 K115Q/K122Q | Small eyes (3/3) Most mutants were pupal lethal or eclosed and died in food |
